# The short- and longer-term effects of brief behavioral parent training versus care as usual in children with behavioral difficulties: study protocol for a randomized controlled trial

**DOI:** 10.1186/s12888-024-05649-8

**Published:** 2024-03-12

**Authors:** Roos S. van Doornik, Saskia van der Oord, Joli Luijckx, Annabeth P. Groenman, Patty Leijten, Marjolein Luman, Pieter J. Hoekstra, Barbara J. van den Hoofdakker, Tycho J. Dekkers

**Affiliations:** 1 Accare Child Study Center, Groningen, The Netherlands; 2 Department of Child and Adolescent Psychiatry, University of Groningen, University Medical Center Groningen, Groningen, The Netherlands; 3 Clinical Psychology, KU Leuven, Louvain, Belgium; 4 Balans, National Parent Association, Bunnik, The Netherlands; 5 Research Institute of Child Development and Education, University of Amsterdam, Amsterdam, The Netherlands; 6 Department of Clinical, Neuro- and Developmental Psychology, Vrije Universiteit Amsterdam, Amsterdam, The Netherlands; 7 Department of Complex Behavioral Disorders and Forensic Youth Psychiatry, Levvel, Amsterdam, The Netherlands; 8 Department of Clinical Psychology and Experimental Psychopathology, University of Groningen, Groningen, The Netherlands; 9 Department of Child and Adolescent Psychiatry, Amsterdam University Medical Center (AUMC), Amsterdam, The Netherlands; 10 Department of Psychology, University of Amsterdam, Amsterdam, The Netherlands

**Keywords:** Children, Behavioral difficulties, Brief, Behavioral parent training, Individually tailored, Psychosocial intervention, Parenting, Randomized controlled trial, Effectiveness, Cost-effectiveness

## Abstract

**Background:**

The access to and uptake of evidence-based behavioral parent training for children with behavioral difficulties (i.e., oppositional, defiant, aggressive, hyperactive, impulsive, and inattentive behavior) are currently limited because of a scarcity of certified therapists and long waiting lists. These problems are in part due to the long and sometimes perceived as rigid nature of most evidence-based programs and result in few families starting behavioral parent training and high dropout rates. Brief and individually tailored parenting interventions may reduce these problems and make behavioral parent training more accessible. This protocol paper describes a two-arm, multi-center, randomized controlled trial on the short- and longer-term effectiveness and cost-effectiveness of a brief, individually tailored behavioral parent training program for children with behavioral difficulties.

**Methods:**

Parents of children aged 2–12 years referred to a child mental healthcare center are randomized to (i) three sessions of behavioral parent training with optional booster sessions or (ii) care as usual. To evaluate effectiveness, our primary outcome is the mean severity of five daily ratings by parents of four selected behavioral difficulties. Secondary outcomes include measures of parent and child behavior, well-being, and parent–child interaction. We explore whether child and parent characteristics moderate intervention effects. To evaluate cost-effectiveness, the use and costs of mental healthcare and utilities are measured. Finally, parents’ and therapists’ satisfaction with the brief program are explored. Measurements take place at baseline (T0), one week after the brief parent training, or eight weeks after baseline (in case of care as usual) (T1), and six months (T2) and twelve months (T3) after T1.

**Discussion:**

The results of this trial could have meaningful societal implications for children with behavioral difficulties and their parents. If we find the brief behavioral parent training to be more (cost-)effective than care as usual, it could be used in clinical practice to make parent training more accessible.

**Trial registration:**

The trial is prospectively registered at ClinicalTrials.gov (NCT05591820) on October 24th, 2022 and updated throughout the trial.

**Supplementary Information:**

The online version contains supplementary material available at 10.1186/s12888-024-05649-8.

## Background

Behavioral difficulties, including oppositional, defiant, aggressive, hyperactive, impulsive, and inattentive behavior, are one of the most common reasons for referral to mental healthcare among children and adolescents [1, 2]. When left untreated, problems may exacerbate and put children at risk of adverse outcomes later on in life, such as school dropout, delinquency, substance use and depression [3, 4]. Ideally, treatment should prevent this escalation of problems, reduce the need for long and intensive treatments, and lower societal costs [5]. Behavioral parent training is the first psychosocial treatment of choice for reducing behavioral difficulties in preschool and school aged children [6–12]. However, its use in clinical practice is limited by a scarcity of certified therapists, long waiting lists, and the typically long and sometimes perceived as rigid nature of behavioral parent training programs, which may lead to parents not starting or finishing behavioral parent training [13–15]. To reduce these barriers, this study evaluates a brief and individually tailored behavioral parent training program for children with behavioral difficulties (the “*PAINT-GGZ*” program, developed by the Psychosocial ADHD & disruptive behavior INTerventions [PAINT] consortium). The brief training includes optional booster sessions for families who need additional support [16].

In behavioral parent training, parents are taught techniques to curtail children’s behavioral difficulties by avoiding or breaking coercive parent–child interactions [17]. Parenting programs have moderate short-term effects on child conduct problems [18], oppositional behavior [19, 20], inattentive and hyperactive/impulsive behavior [21, 22], and parenting [23]. The longer-term effects of behavioral parent training are less consistent: A meta-analysis on behavioral parent training for children with attention deficit/hyperactivity disorder (ADHD) found mostly sustained effects five months to one year after the intervention [24], while a meta-analysis on behavioral parent training for children with disruptive behaviors (e.g., tantrums, arguing, rule-breaking) shows a large heterogeneity in results, with some studies showing sustained effects and other studies showing fade-out effects or sleeper effects three years after the intervention [25]. Overall, the evidence base of behavioral parent training is well-established.

However, a scarcity of certified therapists, long waiting lists, and the typically long and sometimes perceived as rigid nature of behavioral parent training programs undermine the use of parenting interventions in clinical practice. That is, only a minority of families who seek treatment actually receive parent training, because there is a lack of certified therapists who can provide behavioral parent training and waiting lists for parenting interventions are common [15]. Also, clinicians sometimes do not recommend parent training, but rather redirect children towards medication [26]. Additionally, behavioral parent training programs are typically long (8 to 12 sessions [14],) and generally not tailored to the specific behavioral difficulties that parents seek help for [27]. This is problematic, as behavioral parent training programs are more likely to be effective when their content is tailored to the specific problems parents encounter in daily life [14]. The perceived rigidity and length of programs may lead to parents not starting or completing behavioral parent training [15, 28]. That is, at least 25% of parents who are eligible to participate in scientific studies and qualify for behavioral parent training do not enroll in a parenting program and another 26% terminate the training prematurely [13]. Brief and individually tailored behavioral parent training programs may reduce these problems, but the few existing brief programs are barely provided and mainly evaluated in prevention settings [29, 30]. There is thus an urgent need for evidence-based, brief and individually tailored programs.

To address this need, this randomized controlled trial evaluates the effectiveness of a brief and individually tailored behavioral parent training program with optional booster sessions for children with behavioral difficulties. The program is based on intervention elements that were effective in reducing children’s behavioral difficulties in the broader literature and our earlier work: antecedent techniques (i.e., stimulus control techniques) and consequent techniques (i.e., contingency management techniques) [22, 31, 32]. Antecedent techniques are aimed at changing child behavior by manipulating the antecedents of this behavior (e.g., by setting clear rules and providing structure) and consequent behavioral techniques are aimed at changing child behavior by manipulating their consequences (e.g., by praising desired behavior and ignoring undesired behavior). In a recent microtrial in which we evaluated the efficacy of antecedent and consequent techniques, both sets of techniques were found to reduce behavioral problems of children with ADHD in brief (i.e., two sessions) behavioral parent training, of which antecedent techniques were especially effective in decreasing inattention [33]. This microtrial also showed that the magnitude of effects of a brief program (i.e., medium-sized effects) was similar to those of traditional, longer programs on reducing child behavioral difficulties [23, 33, 34].

Building upon the findings of our microtrial, the brief behavioral parent training of the present trial consists of two sessions in which antecedent and consequent techniques are combined and a third session aimed at the evaluation and maintenance of applying the learned techniques. To prevent relapse and the possible use of other, more intensive and expensive treatments, parents can receive booster sessions in the first year after the brief training. Similar to the microtrial, the behavioral parent training is individually tailored by letting parents select four target behaviors they want to address in the training and by creating an individually tailored intervention plan based on the functional analysis (see [33]). A non-randomized pilot study (*N* = 28) preceding the present trial indicated a relatively low dropout rate (14.2%), high treatment feasibility and parent and clinician satisfaction, and a reduction of children’s behavioral difficulties from pre to post intervention [35].

### Objectives and hypotheses

The first objective of this study is to examine the short- and longer-term (i.e., one year) effectiveness of the brief and individually tailored behavioral parent training program with optional booster sessions compared to care as usual (CAU) on daily measured child behavioral difficulties (primary outcome) and on a range of secondary outcomes (see Outcomes). Additionally, we will explore whether a number of general demographic and clinical characteristics, parental attachment, parental psychopathology, parental reward responsivity, and reward and punishment sensitivity of the child moderate the short- and longer-term intervention effects on our primary outcome. The second objective is to investigate the cost-effectiveness of the program compared to CAU. As we assess cost-effectiveness based on measures at baseline and one year after the intervention, we can only draw conclusions about the cost-effectiveness on the longer term.

Based on our earlier work [33, 35], we hypothesize that the brief behavioral parent training with booster sessions will be more effective than CAU in reducing children’s behavioral difficulties. Consequently, we expect parents who receive the brief behavioral parent training to use less subsequent care and thus hypothesize the brief training to be more cost-effective than CAU on the longer term.

## Methods

### Study design

In this two-arm multi-center randomized controlled trial, parents of children who experience behavioral difficulties in the home setting and were referred to a child mental healthcare center, are randomly assigned (1:1 ratio) to either (a) three sessions of brief behavioral parent training with optional booster sessions, or (b) CAU, as regularly provided by the involved mental healthcare centers. There are no restrictions regarding the type (e.g., psychoeducation, parent counseling and support, individual child therapy, medication) or duration of CAU, only the brief parent training investigated in this trial is not allowed. Both parents and children in the brief behavioral parent training group are not allowed to receive CAU until the first posttreatment assessment (T1). After that, CAU is allowed and parents can also make use of booster sessions with the therapist who provided the training.

### Study setting

To ensure our findings reflect the real-world impact of brief behavioral parent training (i.e., to test effectiveness rather than efficacy), the trial is fully embedded in routine mental healthcare. The participating centers are academic and non-academic youth mental healthcare centers of various sizes. Therapists who provide the brief behavioral parent training have a master’s degree in psychology or child, family and education studies and have had postmaster education in cognitive behavioral therapy (at least 50 h) or have a registered higher vocational education degree as social worker in cognitive behavioral therapy. The therapists are trained in the brief parent training by supervisors qualified in cognitive behavior therapy during a one-day training of seven hours, in which therapists practice the parent training by roleplay and evaluate on their performance. Therapists also receive weekly supervision by these qualified supervisors when providing the brief parent training. Additionally, the supervisors provide feedback to each therapist on the audio-taped first session regarding protocol fidelity, intervention integrity, and the process of providing the training (e.g., engaging parents, didactic skills). There are no in- or exclusion criteria for clinicians in the CAU condition. To prevent contamination of the two research arms, therapists never provide both the brief behavioral parent training and CAU to parents participating in this study and are instructed not to share information about the contents of the brief training.

### Stakeholder involvement: parent advisory board

For this trial we have installed a parent advisory board (four members, one of them [co-author JL] represents “*Balans*”, a Dutch association for parents of children with developmental problems). The board is involved in this trial since the grant application. They provided feedback and ideas for the current project and meet biannually in the first and last year of the project and annually in years two, three, and four. In the last year, they will be asked to give their ideas about the interpretation of the results. During the writing of the grant application, the parent advisory board indicated that targeting individual needs is crucial for parents and that booster sessions may be very important and helpful, as some parents may experience relinquishing of their parenting skills after finishing behavioral parent training (e.g., in stressful times). The board also tested the suitability of the measures: they considered the time investment of the questionnaires to be acceptable and indicated to use audiotapes rather than videotapes as observational measurement, as parents may perceive audiotapes as less invasive and interfering with their privacy. The members of the board receive reimbursements for all meetings.

### Eligibility criteria

Families eligible for the trial must meet the following inclusion criteria:

- The child is aged between 2 and 12 years;
- Parents have to identify at least four behavioral difficulties of the child that occur in the home setting and that they want to target in the training, using an adapted version of a list of target behaviors [33, 36]. This list contains 29 behaviors that can be targeted in the training, such as hyperactive, impulsive, inattentive, oppositional and defiant behavior. The items are derived from target problems that parents mentioned in previous behavioral parent training groups and concern child behaviors that are commonly targeted in behavioral parent training in clinical practice, confirming ecological validity [36].

Exclusion criteria are:

- The child uses psychotropic medication (currently or in the month before the screening);
- The child has at any time received a diagnosis of autism spectrum disorder (ASD) in clinical practice, as (parents of) children with ASD may have different needs and therefore may require different treatments than children with behavioral difficulties without ASD;
- The child has a known IQ-score below 70, as (parents of) children with intellectual disabilities may have different needs and therefore may require different treatments than children with behavioral difficulties and typical intellectual abilities;
- Parents received behavioral parent training aimed at reducing the behavioral difficulties of the concerned child in the year prior to the start of the study;
- It is not a suitable period for the parents and/or the child to participate in the study (e.g., moving, divorce);
- The child is not living in the same household as the parent(s) who participate(s) in the trial during at least four weekdays (to ensure that our primary outcome can be reported by the same informant(s) and that parents can apply the intervention plans at home).

### Interventions

The parent training program includes the behavioral techniques that were identified as effective for reducing behavioral difficulties (i.e., oppositional, defiant, aggressive, hyperactive, impulsive, and inattentive behavior) in a preceding microtrial [33] and in the broader literature [22, 23, 31, 32, 34]. Importantly, all behavioral techniques are explained to parents and based on a functional analysis, the most suitable techniques are selected and tailored to the specific problem behaviors that parents experience with their child and to the home context. Prior to the brief training, with a researcher parents choose four daily occurring target behaviors from the list of target behaviors (see Primary Outcome) that they prefer to work on in the training [33, 36, 37]. For each of the four behaviors, parents also indicate in which situation the behavior occurs, using the situations that are included in the Home Situation Questionnaire [37]. The therapist providing the brief training receives a list of the chosen four behaviors in specific situations before the training.

In the first session, parents are briefly educated about supposed underlying mechanisms of behavioral difficulties in children. They learn that children with behavioral difficulties may have problems in executive functioning, such as inhibition or working memory, which may influence the way they process environmental information and exert control over their behavior [38, 39]. Parents are taught how they can use antecedent techniques (i.e., setting rules, giving clear instructions, structuring situations, discussing situations in advance) to support their child’s executive functioning and elicit more desirable and prosocial behaviors. Parents are also taught that children with behavioral difficulties may show altered sensitivity to reinforcement and punishment, which may influence how children learn from rewards and negative consequences and the way in which their behavior is shaped by environmental consequences as provided by parents [40–44]. Their altered reinforcement sensitivity may lower the likelihood that children change behavior in response to corrective reactions and result in behaviors that are reward-oriented on the short term [40, 43]. The therapist explains that consequent techniques (i.e., reinforcement [e.g., praise], planned ignoring, non-violent discipline techniques [e.g., correction, natural consequences]) are important to deal with these sensitivities, while emphasizing that the majority of the used consequent techniques should concern reinforcement. In their explanations, therapists also stress parents that the psycho-education is based on research involving many children and could therefore not directly apply to the situation of their child. After this psycho-education, the parents and therapist select a first target problem behavior along with the specific situation in which this behavior occurs daily. This behavior is chosen from the four behaviors in specific situations that parents selected beforehand with a researcher to target in the training. Together, the parents and therapist make a functional analysis of the selected behavior and create an individually tailored intervention plan. This plan contains the most suitable of the four antecedent techniques and one consequent technique (i.e., praise). The techniques are selected according to the functional analysis and adapted to the abilities and needs of the child and parents. Parents write down their intervention plan with the therapist and receive a handout with explanations of the techniques. Directly after each session, parents carry out their intervention plan at home the next week.

In the second session, the first plan is evaluated and adapted if necessary. The parents and therapist briefly recapitulate the psycho-education and intervention plan of the first session and discuss a second target problem behavior in the same way as the first problem behavior. They make an individually tailored intervention plan for the second target behavior, which again includes the most suitable techniques of all discussed antecedent and consequent techniques, based on the functional analysis of the second target behavior. Based on the results of our microtrial that antecedent techniques are effective sooner after the training (i.e., immediately) and are more effective in decreasing symptoms of inattention than consequent techniques [33], the intervention plan of the first session mainly contains antecedent techniques, while the intervention plan of the second session includes both antecedent and consequent techniques. The consequent techniques always include forms of reinforcement (e.g., praise, rewards) and can optionally include ignoring or mild negative consequences. In the third session, the second intervention plan is evaluated and adapted if necessary. Maintenance training is provided by encouraging parents to think about how the techniques can be used for other problems. Furthermore, during this session parents rehearse and practice designing an intervention plan accordingly for possible new behavior problems.

In the optional booster sessions, parents can discuss either the same or new target behaviors and create or adapt an intervention plan with the antecedent and consequent techniques that are taught in the earlier sessions. Parents are stimulated by the therapist to use the steps they have learned in the earlier sessions. Parents implement new or adapted intervention plans directly after the session. Booster sessions are not meant as prolongation of the brief training, but as stand-alone sessions to support parents in refreshing and maintaining to apply the learned techniques. Booster sessions can be combined with any CAU.

Parents in the control arm receive CAU, which in Dutch mental healthcare typically consists of psychoeducation, parent counseling and support, medication, longer behavioral parent training programs, family therapy, support at school, and/or cognitive behavior therapy or other therapy or support for the child.

### Treatment fidelity

Intervention fidelity (i.e., the percentage of items covered in each session) is assessed with a fidelity checklist using an adapted version of the procedures of Abikoff and colleagues [45, 46]. This implies that all intervention sessions (including the booster sessions) are audiotaped. The audiotapes of 20% randomly selected sessions will be scored on intervention fidelity by independent evaluators. After each session, therapists also have to complete a fidelity checklist in which they are asked which items they covered.

### Recruitment

Parents of children with behavioral difficulties who are referred to one of the participating Dutch mental healthcare centers are recruited by clinicians within these centers. After the diagnostic assessment of the child and before any treatment has started, the clinicians involved in the diagnostic assessments inform parents of children who seem eligible about the study and hand out an information letter. Clinicians provide researchers with contact information of parents that have consented to sharing information and are interested in participation. The researchers then further inform parents about the trial through telephone contact. If parents want to participate, the researchers ask both parents, or other legal caretakers of the child, to provide informed consent. By default, parents have 14 days to decide upon participation and ask the researchers any questions. If needed, parents can ask for more time to decide upon participation. Once parents have provided consent, the researchers screen whether families are eligible for the study and inform parents whether they can participate.

### Allocation to study arms

Randomization is performed with an online generated randomization schedule [47], using blocks of six participants for each healthcare center to ensure equal allocations to both arms within and across centers. The randomization schedule is only known and administered by independent researchers who are not involved in the trial in any other way. When participants can be randomized, the independent researchers are informed at which healthcare center the parents are participating and asked to inform the responsible researchers about which condition has been randomly assigned to these parents. Randomization occurs directly after the baseline measurement (T0).

### Procedure

Parents in the brief behavioral parent training arm receive three sessions of behavioral parent training. The first two sessions take 120 min each and are planned one or two weeks apart. The third session takes 60 min and is planned one week after the second session. To take parents’ and therapists’ availability into account, some flexibility in planning is allowed as long as all three sessions are planned within six weeks with at least one week between two consecutive sessions. After the third session, parents wishing to receive additional support can receive CAU and/or booster sessions. The booster sessions take 60 min each and can be provided maximally once every four weeks up to one year after the last session of the brief behavioral parent training program. Booster sessions can be suggested by the therapist who provided the brief behavioral parent training or by other involved clinicians, or requested by parents themselves.

**Fig. 1:**
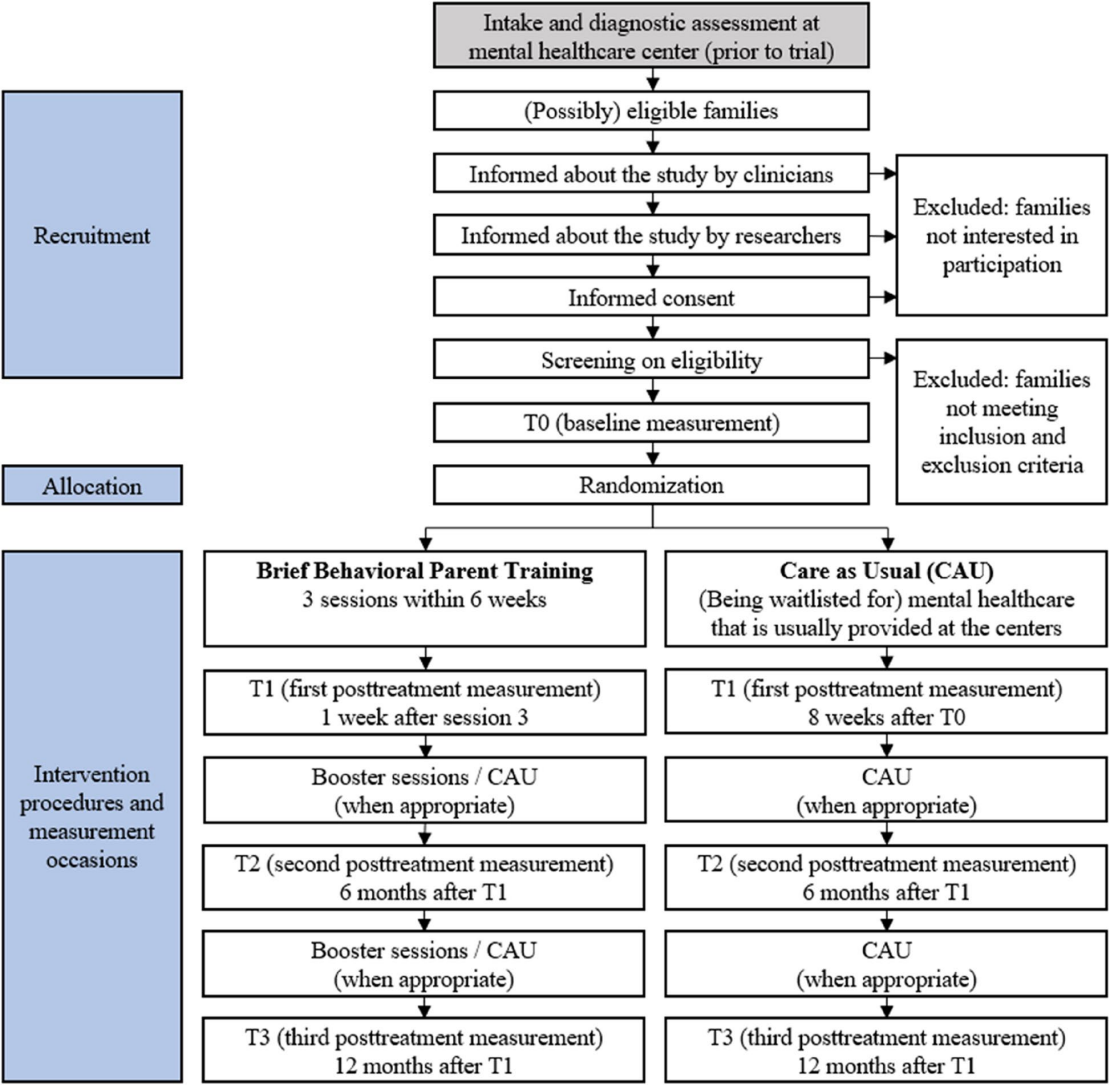
Participant Timeline

The trial contains four measurement occasions, see Fig. 1 for the participant timeline. T0 takes place after parents provide consent and are found eligible for participation, before randomization. The first posttreatment measurement (T1) takes place one week after the third session for parents in the brief behavioral parent training arm and eight weeks after T0 for parents in the CAU arm. The second posttreatment measurement (T2) takes place six months after T1 and the third posttreatment measurement (T3) takes place twelve months after T1. Participation in the trial therefore takes approximately 14 months in total. The care families receive at the child mental healthcare center may terminate sooner if they do not need or want care anymore.

The outcomes are measured with questionnaires, audiotapes, and short daily phone calls. Completing the questionnaires (including those measuring moderator variables) takes 30 to 45 min per measurement occasion, making the audiotapes takes about 30 min per measurement occasion (i.e., two recordings of 15 min each), and making the phone calls takes two to three minutes each. Furthermore, parents in the behavioral parent training arm fill out the ECBI-I questionnaire at the beginning of and two weeks after each booster session, which takes a couple of minutes.

### Demographic information and clinical characteristics

Parents will provide information on children’s age and gender, on their own and children’s ethnicity, educational background, and household composition, and on children’s previous and ongoing pharmacological and non-pharmacological treatments, medical conditions, and clinical diagnoses (e.g., ADHD, oppositional defiant disorder [ODD], conduct disorder [CD]).

### Outcomes

#### Primary outcome

##### Individually determined daily ratings of behavioral difficulties

The primary outcome is the mean severity of parents’ daily ratings of four selected target behaviors in specific home situations. On the adapted version of the list of target behaviors [33, 36], parents indicate whether the 29 behaviors occur daily (*yes/no*). For the behaviors scored as yes, parents rate the severity on a 5-point Likert scale ranging from 1 (*not severe*) to 5 (*extremely severe*). Behaviors scored as no are coded 0. With a researcher, parents choose four daily occurring target behaviors from this list that they prefer to work on in the training. Parents also indicate in which situation these behaviors occur, using the situations that are included in the Home Situation Questionnaire [37].

For each measurement occasion, during preferably five but at least four consecutive weekdays, short daily phone calls with parents are made to evaluate whether the four selected target behaviors occurred in the past 24 h in the selected situation (*yes/no*). Items scored as no are rated 0 and items scored as yes are rated on a 5-point Likert scale ranging from 1 (*not severe*) to 5 (*extremely severe*). This outcome is measured at all timepoints (T0-T3). For each timepoint, the average score of all four behaviors on all weekdays is used as outcome measure.

#### Secondary outcomes

##### Parent-reported behavioral difficulties

Children's behavioral difficulties are assessed with the Intensity scale of the Eyberg Child Behavior Inventory (ECBI-I) [48]. The Intensity scale consists of 36-items for parents of children aged 2 to 16 and measures the frequency of specific problem behavior on a 7-point Likert scale from 1 (*never*) to 7 (*always*). The convergent and divergent validity and the reliability of the ECBI-I are well established [49]. This outcome is measured at all timepoints, and at the beginning of and two weeks after each booster session for parents who receive the brief behavioral parent training.

##### Child well-being

Child well-being is assessed with the Health-Related Quality of Life Questionnaire (KINDL-R) [50]. Parents rate their children's quality of life on 20 items regarding emotional well-being, self-esteem, family functioning, social contacts, and school, of which the total score will be used. Parents rate the items on a Likert scale ranging from 1 (*never*) to 5 (*all the time*). The KINDL-R has revealed sufficient internal consistency (α = 0.82) [51]. This outcome is measured at all timepoints.

##### Parenting behaviors

Parenting behaviors are assessed with the Alabama Parenting Questionnaire (APQ; [52]). The APQ is a 42-item parent-report measure assessing five categories of parenting practices (involvement, positive parenting, poor monitoring/supervision, inconsistent discipline, and corporal punishment), of which the total score will be used. Parents rate their parenting on a 5-point scale ranging from 1 (*never*) to 5 (*always*), with higher scores representing higher levels of the particular parenting category. The reliability and validity of the APQ are well established [52]. This outcome is measured at all timepoints.

##### Observed parent and child behaviors

To include an observational measurement of parent and child behaviors, parents are asked to audio record their mealtime routines for at least 15 min on two different weekdays. Mealtimes are notoriously busy times in family lives and thus well suited as a setting for an ecologically valid measure. The recordings of mealtime routines are a masked measure (i.e., assessors are not aware of the intervention condition), based on the method that was used by Herbert et al. [53]. Using the recordings, the following behaviors will be scored with a global coding system: parental behavior (both supportive and non-supportive parenting), child misbehavior, and emotional talk. A subsample of recordings will be double coded until sufficient interrater reliability is established. This outcome is measured at all timepoints except T2, to minimize the burden for families.

##### Parenting stress

Parenting stress is assessed with the Parental Stress Scale (PSS; [54]). The PSS is an 18-item parent report scale that measures positive (e.g., emotional benefits) and negative (e.g., restrictions) aspects of parenting, of which the total score will be used. Parents have to agree or disagree with statements concerning parenting on a 5-point scale ranging from 1 (*strongly disagree*) to 5 (*strongly agree*). The adequate reliability (α = 0.83) and validity of the PSS have been demonstrated [54]. This outcome is measured at all timepoints.

##### Parenting self-efficacy

Parenting self-efficacy is measured with the subscale Efficacy of the Parenting Sense of Competence Scale (PSOC; [55]). On the eight items of this subscale, parents rate their capability level and problem-solving ability regarding their parental role on a 6-point scale, ranging from 1 (*strongly disagree*) to 6 (*strongly agree*). The internal consistency (α = 0.76) of the subscale Efficacy has been established [55]. This outcome is measured at all timepoints.

##### Quality of the parent–child relationship

The quality of the parent–child relationship is measured with the parent version of the Parent–Child Interaction Questionnaire-Revised (PACHIQ-R; [56]). On 21 items, parents rate their relationship with their child on a 5-point scale, ranging from 1 (*strongly disagree*) to 5 (*strongly agree*), of which the total score will be used. The PACHIQ-R has been demonstrated to have high internal consistency (ranging between α = 0.79 and α = 0.93) [56]. This outcome is measured at all timepoints.

##### Utilities

Utilities, also called preferred health states, are assessed using quality-adjusted life years (QALYs). QALYs range between 0 and 1, where 0 represents death and 1 perfect health. QALYs are calculated based on the EuroQol-5D-5L (EQ-5D) questionnaire [57, 58], which parents fill out about their child. The EuroQol-5D-5L measures the child’s health with five items (mobility, self-care, daily activities, pain, and anxiety/depression) on 5-level categorical scales. EQ-5D responses will be transferred into QALYs based on the Dutch EQ-5D-5L tariff for adults [59], as a tariff for Dutch children is not yet available. The content and face validity of the EuroQol-5D-5L are well established [58]. This outcome is measured at T0 and T3.

##### Use of healthcare

Use of mental healthcare within the organization where the child is treated is measured by drawing up an inventory (based on patient records) of the type of care (brief behavioral parent training, booster sessions, CAU) that is used and the duration (in minutes) of this care between T0 and T3 in both arms. The broader use of healthcare within and outside the mental healthcare centers is assessed with the *Vragenlijst Intensieve Jeugdzorg*, a Dutch questionnaire on intensive youth care [60]. This questionnaire assesses the use of a wide variety of healthcare (e.g., contact with general practitioner or medical professionals, use of social services or (alternative) medicines), along with the child’s use of education, contact with judicial authorities, and losses in the productivity of parents. Parents complete this questionnaire about the use and intensity of healthcare of both the child and themselves in the past three months. This outcome is measured at T0 and T3. Both measures of healthcare use (i.e., inventory of care and questionnaire) will be combined to obtain a complete image of all used care.

##### Costs of healthcare

Healthcare costs are estimated from a societal perspective [5]. Costs of mental healthcare are estimated by multiplying the used care by the reference prices provided in the Cost Manual of the Dutch National Health Care Institute. Costs of medication are estimated by using prices provided by the Dutch National Health Care Institute [61]. Costs in other sectors (e.g., education, justice) are estimated by using reference prices provided in the Manual Intersectoral Costs and Benefits [62]. All costs are estimated after families’ participation in the study, when their healthcare use is fully measured.

#### Candidate moderators

In addition to the general demographic and clinical characteristics and some baseline characteristics such as the severity of behavioral problems, the following variables are included to explore whether they moderate the short- (T1) and longer-term (T2, T3) intervention effects: parental attachment as measured with the 12-item version of the Revised Experiences in Close Relationship questionnaire (ECR-R; [63, 64]),parental psychopathology as measured with the 25 items of the Strengths and Difficulties Questionnaire (SDQ) for adults [65], parental reward responsivity as measured with the 8-item Reward Responsiveness (RR) questionnaire [66], and child reward responsivity and punishment sensitivity as measured with the Punishment Sensitivity and Reward Responsivity subscales (combined 22 items) of the Sensitivity to Punishment and Sensitivity to Reward Questionnaire for Children (SPSRQ-C; [67]). All candidate moderators are measured at T0 only and reported by parents.

#### Parent and therapist satisfaction

##### Parents’ satisfaction with the brief behavioral parent training

Satisfaction with the brief behavioral parent training of parents who received the intervention are measured in two ways. First, parents are asked to fill out a self-developed satisfaction questionnaire, which is based on questions of the Parent Satisfaction Questionnaire [68], the Therapy Attitude Inventory [69], and the satisfaction questionnaire that was used in Breider et al. [70]. Parents who received the brief behavioral parent training answer 13 questions about their satisfaction with the brief behavioral parent training at T1 and three questions about their satisfaction with the booster sessions (if used) at T3 on a 5-point scale ranging from 1 (*strongly disagree*) to 5 (*strongly agree*). Parents also grade the brief behavioral parent training generally between 1 (*very bad*) and 10 (*excellent*). Second, one or multiple focus group(s) will be organized with a small number of parents. In the focus group(s), the new program will be qualitatively evaluated and information about feasibility and barriers and facilitators for the implementation of the training in clinical settings will be gathered. The focus group(s) will be held after the inclusion of parents is finished, which is anticipated to be at the beginning of 2025.

##### Therapists’ satisfaction with the brief behavioral parent training

Therapists' satisfaction and opinion about the intervention are measured in two ways. First, therapists will be asked to fill in a self-developed satisfaction questionnaire, which is based on questions of the Parent Satisfaction Questionnaire [68], the Therapy Attitude Inventory [69], and the satisfaction questionnaire that was used in Breider et al. [70]. Therapists have to answer seven questions on a 5-point scale ranging from 1 (*strongly disagree*) to 5 (*strongly agree*) and give the brief behavioral parent training a general grade between 1 (*very bad*) and 10 (*excellent*). Second, one or multiple focus group(s) will be organized with a selection of therapists from different healthcare centers who provided the brief parent training and range in years of work experience and to how many families they have provided the brief behavioral parent training. In the focus group(s), important barriers and facilitators for the implementation of the program will be identified. Both outcomes will be measured after the trial is finished, which is anticipated to be at the beginning of 2026.

#### Adverse reactions

Adverse reactions are measured with a self-developed questionnaire. These reactions involve negative experiences of parents and/or children that are, according to parents, related to the care they received either in the brief parent training treatment arm or in the CAU arm. The questionnaire consists of six questions that ask parents whether they experience a decline in their trust in healthcare, (anxiety of) negative reactions from others (i.e., stigma), mental health issues, difficulties in relationships and activities, or any other difficulties or concerns that could be related to the care they received during the trial. For each question that applies to them, we ask parents to shortly elaborate on their experiences and indicate when these experiences happened. These answer options are based on the Medical and Psychological Events and Difficulties (MAPED) questionnaire that was used in the Online Parent Training for The Initial Management of ADHD referrals (OPTIMA) trial [71]. This outcome is measured at T3.

### Sample size calculation

Based on our previous study that found medium-sized short term effects on daily ratings of behavioral difficulties compared to waitlist (range of Cohen’s *d* = 0.35 to *d* = 0.66, [33]) and studies showing medium-sized parenting intervention effects on behavioral difficulties using parent-report rating scales [19, 20, 25, 36, 72], we estimate the effect of our brief behavioral parent training program as compared to CAU to be at least small (*d* = 0.25).

A power analysis has been performed using G*Power software [73]. Based on an effect size of *d* = 0.25 (*f* = 0.125), two groups and three repeated measures (T0, T1, T2 for short term effectiveness), with *r* = 0.60 between-measurement relation, a power of 0.80 and α = 0.05, we need at least 42 participants per group, resulting in a total of 84 participants. Given that the data are clustered, we increased the sample size with 10% [74], resulting in a total of 93 participants. This is in line with the number of participants in similar studies comparing behavioral treatments to CAU (e.g., [36]).

### Statistical analysis

Data will be analyzed on an intention-to-treat basis. To examine the effects of the intervention on the primary and secondary outcomes, we will conduct multilevel analyses (mixed modeling), taking missing data into account [75]. Depending on the fit of the models, up to four hierarchical levels will be distinguished: outcomes (level 1), nested within children (level 2), nested within therapists (level 3), nested within healthcare centers (level 4). Random intercepts at the subject level, therapist level and healthcare center level will be included only if the Likelihood Ratio Test shows a significant improvement of the model fit. Time will be included as short-term (T1) and longer-term (T2, T3) within-subjects factors and condition (brief parent training vs. CAU) as between-subjects factor. The interaction between time and condition will be examined to interpret changes over time between conditions. To examine whether the candidate moderator variables moderate the intervention effects, these variables will also be added as interaction effects to the multilevel analyses.

To assess the cost-effectiveness of the brief behavioral parent training compared to CAU, we will calculate the Incremental Cost Effectiveness Ratio (ICER) from a societal perspective. We will perform both a cost-utility analysis (based on QALYs) and cost-effectiveness analyses (based on the daily ratings of behavioral difficulties and based on ECBI-I scores). An ICER is calculated by dividing the difference in the total costs by the difference in the total effect (either QALYs or the daily ratings of behavioral difficulties and ECBI-I scores, respectively). In these analyses, the costs of healthcare will be used and interpolated to the previous 12 months. We will compare each ICER based on relevant willingness to pay thresholds. Bootstrapping will be used to calculate the reliability of our estimates.

### Trial duration

October 2022 – December 2026.

### Data management

Digital data, both raw and processed data are stored at a study specific secured folder within the network of Accare, the institution at which the research is carried out. This study specific folder can only be accessed by authorized personnel, who are involved in this study. All questionnaire data and processed audio files are stored in one, pseudonymized file, that is accompanied by a 'readme' text file that contains a code book explaining the meaning of all variables. A separate logbook-file will be created documenting all decisions that are made during the process from raw to processed data. Raw data containing identifiable information is kept strictly separate from the processed data and can only be accessed by two of the supervisors (BJvdH, TJD), research assistants and PhD candidate (RSvD). After the research project will be completed (i.e., data collection, data analysis and publishing of research articles), all digital data will be transferred to a study specific folder for long-term storage. The folder can only be accessed by authorized personnel. All research data will be stored for at least 15 years after the data collection has been completed. Upon request, the processed, pseudonymized data will be available for fellow researchers and made available (restricted access) for use in future research projects, to which all participants have consented before enrolling in the study. Requests for re-use of data will be evaluated to check whether the research question falls within the scope of the informed consent.

### Ethics and dissemination

This study has been granted a non-WMO statement from the Medical Ethics Review Committee (METc) of the University Medical Center Groningen, meaning that the study does not meet the conditions of the Medical Research Involving Human Subjects Act. Ethics approval has been obtained from the Scientific and Ethical Review Board (VCWE) of the Vrije Universiteit (VU) Amsterdam (VCWE-2022–124). Approval to participate in this study has been obtained from all research partners. The results of this study will be submitted for publication in peer-reviewed journals. In addition, findings will be presented at scientific conferences and shared with stakeholders (e.g., parents, clinicians), for instance on our website (https://www.paint-studies.nl/) and social media channels. Regarding clinical practice, findings will be shared with parents and mental healthcare institutions, partly in collaboration with Balans (e.g., vlogs, flyers, presentations, newsletters). If the brief behavioral parent training with booster sessions is more effective than CAU in reducing children’s behavioral difficulties, the brief training can be implemented in clinical practice.

## Discussion

This study protocol describes a multi-center randomized controlled trial investigating the short- and longer-term effectiveness and the cost-effectiveness of a brief, individually tailored behavioral parent training program that aims to reduce children’s behavioral difficulties. Although behavioral parent training is an evidence-based psychosocial treatment for reducing children’s behavioral difficulties, few families currently receive the intervention. This may be due to, among other reasons, a shortage of certified therapists, long waiting lists, and a perceived rigidity and length of behavioral parent training programs. Brief and individually tailored parenting interventions may reduce these problems and could therefore be a promising way to make behavioral parent training more accessible for parents.

We anticipate multiple challenges that the trial could face. For instance, clinicians’ policies and attitudes towards parent training may complicate the enrollment of eligible families in clinical practice [26]. Clinicians might be used to their work routines and experience resistance or difficulty getting acquainted with the trial, for instance because they do not feel sufficiently knowledgeable about the contents and effects of the brief behavioral parent training or feel uncertain about the procedures and administration that come with families’ participation in the trial, or because families may express hesitation about participating in a trial. As a result, clinicians may not refer eligible families or (intuitively) recommend usual care (e.g., other parent training programs or parent support, child treatments, medication) rather than the trial. We aim to tackle this hindrance by making clinicians aware that participation in this trial is in line with the stepped-care approach as delineated in international and Dutch treatment guidelines for children with ADHD and for children with disruptive behavior disorders, which suggest offering non-pharmacological treatment that includes psycho-education and practical advices, such as the brief behavioral parent training, before starting more intensive treatment or medication [6, 8, 10, 12].

Other challenges concern potential hindrances for parents to participate. For instance, parents might find it difficult to complete all measurements during four occasions over an extended period of time (i.e., approximately 14 months). For several reasons, it can be difficult to stay dedicated to participate (e.g., motivational issues, time constraints). Besides, parents presumably take part in the trial hoping to receive the brief parent training and might therefore be disappointed when they are randomized into the CAU arm, which can cause their motivation for participation to decline. This might make parents who receive CAU more likely to drop out of the study. We anticipate this challenge by keeping parents involved in the measurements through frequent reminders, expressing the value of parents’ participation and thanking parents after each completed measurement. We also offer parents who consider quitting the study options to reduce the burden of the measurements (e.g., four instead of five daily phone calls, making less or no audio recordings) to possibly keep them involved for a longer period of time.

The results of the trial could have meaningful societal implications for children with behavioral difficulties and their parents. If we find the brief behavioral parent training to be more (cost-)effective than CAU, the treatment could be used in clinical practice to make parent training more accessible.

Quotient; EQ-5D-5L: EuroQol-5 Dimension 5-Level; KINDL-R: Health-Related Quality of Life Questionnaire (German abbreviation); MAPED: Medical and Psychological Events and Difficulties; METc: Medical Ethics Review Committee; ODD: Oppositional Defiant Disorder; PACHIQ-R: Parent–Child Interaction Questionnaire-Revised; PSOC: Parenting Sense of Competence Scale; PSS: Parental Stress Scale; QALYs: quality-adjusted life years; RR: Reward Responsiveness Questionnaire; SDQ: Strengths and Difficulties Questionnaire; SPSRQ-C: Sensitivity to Punishment and Sensitivity to Reward Questionnaire for Children; VCWE: Scientific and Ethical Review Board; V31, U: Vrije Universiteit Amsterdam.

### Supplementary Information

**Supplementary Material 1. **

**Supplementary Material 2. **

**Supplementary Material 3. **

## Data Availability

No datasets were generated or analysed during the current study.

## Abbreviations

ADHD: Attention-Deficit/Hyperactivity Disorder
APQ: Alabama Parenting Questionnaire
ASD: Autism Spectrum Disorder
CAU: Care as usual
CD: Conduct Disorder
ECBI-I: Eyberg Child Behavior Inventory Intensity scale
ECR-R: Experiences in Close Relationship Questionnaire Revised
EQ-5D-5L: EuroQol-5 Dimension 5-Level
ICER: Incremental Cost Effectiveness Ratio
IQ: Intelligence Quotient
KINDL-R: Health-Related Quality of Life Questionnaire (German abbreviation)
MAPED: Medical and Psychological Events and Difficulties
METc: Medical Ethics Review Committee
ODD: Oppositional Defiant Disorder
PACHIQ-R: Parent-Child Interaction Questionnaire-Revised
PSOC: Parenting Sense of Competence Scale
PSS: Parental Stress Scale
QALYs: Quality-adjusted life years
RR: Reward Responsiveness Questionnaire
SDQ: Strengths and Difficulties Questionnaire
SPSRQ-C: Sensitivity to Punishment and Sensitivity to Reward Questionnaire for Children
VCWE: Scientific and Ethical Review Board
VU: Vrije Universiteit Amsterdam

## References

[1] Antshel KM (2015). Psychosocial Interventions in Attention-Deficit/Hyperactivity Disorder. Child and Adolesce Psychiatr Clin N Am.

[2] Polanczyk GV, Salum GA, Sugaya LS, Caye A, Rohde LA (2015). Annual Research Review: A meta-analysis of the worldwide prevalence of mental disorders in children and adolescents. J Child Psychol Psychiatry.

[3] Franke B, Michelini G, Asherson P, Banaschewski T, Bilbow A, Buitelaar JK, Cormand B, Faraone SV, Ginsberg Y, Haavik J, Kuntsi J, Larsson H, Lesch K-P, Ramos-Quiroga JA, Réthelyi JM, Ribases M, Reif A (2018). Live fast, die young? A review on the developmental trajectories of ADHD across the lifespan. Eur Neuropsychopharmacol.

[4] Reef J, Diamantopoulou S, van Meurs I, Verhulst FC, van der Ende J (2011). Developmental trajectories of child to adolescent externalizing behavior and adult DSM-IV disorder: results of a 24-year longitudinal study. Soc Psychiatry Psychiatr Epidemiol.

[5] Drummond MF, Sculpher MJ, Claxton K, Stoddart GL, Torrance GW (2015). Methods for the economic evaluation of health care programmes.

[6] Akwa GGZ. Zorgstandaard ADHD [Dutch ADHD guidelines]. 2019. Retrieved December 2023 from https://www.ggzstandaarden.nl/zorgstandaarden/adhd/samenvatting.

[7] Brestan  EV, Eyberg SM (1998). Effective psychosocial treatments of conduct-disordered children and adolescents: 29 years, 82 studies, and 5,272 kids. J Clin Child Psychol.

[8] National Institute for Health and Care Excellence. Antisocial behaviour and conduct disorders in children and young people: recognition and management (NICE guideline 158). 2017. Retrieved December 2023 from www.nice.org.uk/guidance/cg158. 32073810

[9] Eyberg SM, Nelson MM, Boggs SR (2008). Evidence-based psychosocial treatments for children and adolescents with disruptive behavior. J Clin Child Adolesc Psychol.

[10] Nederlandse Vereniging voor Psychiatrie. Oppositionele stoornis en gedragsstoornis. 2013. Retrieved December 2023 from https://richtlijnendatabase.nl/richtlijn/oppositionele_stoornis_en_gedragsstoornis/oppositionele_stoornis_-_startpagina.html.

[11] Kaminski JW, Claussen AH (2017). Evidence Base Update for Psychosocial Treatments for Disruptive Behaviors in Children. J Clini Child Adolesc Psychol.

[12] National Institute for Health and Care Excellence. Attention deficit hyperactivity disorder: Diagnosis and management (NICE guideline 87). 2019. Retrieved December 2023 from https://www.nice.org.uk/guidance/ng87 . 29634174

[13] Chacko A, Jensen SA, Lowry LS, Cornwell M, Chimklis A, Chan E, Lee D, Pulgarin B (2016). Engagement in Behavioral Parent Training: Review of the Literature and Implications for Practice. Clin Child Fam Psychol Rev.

[14] Evans SW, Owens JS, Wymbs BT, Ray AR (2018). Evidence-Based Psychosocial Treatments for Children and Adolescents With Attention Deficit/Hyperactivity Disorder. J Clin Child Adolesc Psychol.

[15] Weisenmuller C, Hilton D (2021). Barriers to access, implementation, and utilization of parenting interventions: Considerations for research and clinical applications. Am Psychol.

[16] Hoekstra PJ, van den Hoofdakker BJ, Rosenau PT, Dietrich A, Leijten P, Groenman AP (2023). We need better long-term intervention programs in mental health care for children and young people with chronic vulnerabilities. Eur Child Adolesc Psychiatry.

[17] McMahon RJ, Forehand RL (2003). Helping the Noncompliant Child: Family-based treatment for oppositional behavior.

[18] Leijten P, Scott S, Landau S, Harris V, Mann J, Hutchings J, Beecham J, Gardner F (2020). Individual Participant Data Meta-analysis: Impact of Conduct Problem Severity, Comorbid Attention-Deficit/Hyperactivity Disorder and Emotional Problems, and Maternal Depression on Parenting Program Effects. J Am Acad Child Adolesc Psychiatry.

[19] Bakker MJ, Greven CU, Buitelaar JK, Glennon JC (2017). Practitioner Review: Psychological treatments for children and adolescents with conduct disorder problems - a systematic review and meta-analysis. J Child Psychol and Psychiatry.

[20] McCart MR, Priester PE, Davies WH, Azen R (2006). Differential Effectiveness of Behavioral Parent-Training and Cognitive-Behavioral Therapy for Antisocial Youth: A Meta-Analysis. J Abnorm Child Psychol.

[21] Daley D, van der Oord S, Ferrin M, Cortese S, Danckaerts M, Doepfner M, van den Hoofdakker BJ, Coghill D, Thompson M, Asherson P, Banaschewski T, Brandeis D, Buitelaar J, Dittmann RW, Hollis C, Holtmann M, Konofal E, Lecendreux M, Rothenberger A, Sonuga-Barke EJ (2017). Practitioner review: Current best practice in the use of parent training and other behavioural interventions in the treatment of children and adolescents with attention deficit hyperactivity disorder. J Child Psychol Psychiatry.

[22] Hornstra R, Dekkers TJ, Bosmans G, van den Hoofdakker B, van der Oord S (2022). Attachment Representation Moderates the Effectiveness of Behavioral Parent Training Techniques for Children with ADHD: Evidence from a Randomized Controlled Microtrial. Res Child Adolesc Psychopathology.

[23] Dekkers TJ, Groenman AP, Wessels L, Kovshoff H, Hoekstra PJ, van den Hoofdakker BJ (2022). Which factors determine clinicians’ policy and attitudes towards medication and parent training for children with Attention-Deficit/Hyperactivity Disorder?. Eur Child Adolesc Psychiatry.

[24] Doffer DPA, Dekkers TJ, Hornstra R, van der Oord S, Luman M, Leijten P, Hoekstra PJ, van den Hoofdakker BJ, Groenman AP. Sustained Improvements by Behavioural Parent Training for Children with Attention-Deficit/Hyperactivity Disorder: A Meta-Analytic Review of Longer-Term Child and Parental Outcomes. 2023. Retrieved December 2023 from https://psyarxiv.com/cymva/. 10.1002/jcv2.12196 PMC10501699 37720584

[25] Van Aar J, Leijten P, Orobio de Castro B, Overbeek G. Sustained, fade-out or sleeper effects? A systematic review and meta-analysis of parenting interventions for disruptive child behavior. Clin Psychol Rev. 2017;51:153–63. 10.1016/j.cpr.2016.11.006. 10.1016/j.cpr.2016.11.006 27930935

[26] Dekkers TJ, Hornstra R, van der Oord S, Luman M, Hoekstra PJ, Groenman AP, van den Hoofdakker BJ (2022). Meta-analysis: Which Components of Parent Training Work for Children With Attention-Deficit/Hyperactivity Disorder?. J Am Acad Child Adolesc Psychiatry.

[27] DuPaul GJ, Evans SW, Mautone JA, Owens JS, Power TJ (2020). Future Directions for Psychosocial Interventions for Children and Adolescents with ADHD. J Clin Child Adolesc Psychol.

[28] Joseph HM, Farmer C, Kipp H, Kolko D, Aman M, McGinley J, Arnold LE, Gadow KD, Findling RL, Molina BSG (2019). Attendance and engagement in parent training predict child behavioral outcomes in children pharmacologically treated for attention-deficit/hyperactivity disorder and severe aggression. J Child Adolesc Psychopharmacol.

[29] Kolko DJ, Lindhiem O (2014). Introduction to the Special Series on Booster Sessions and Long-Term Maintenance of Treatment Gains. J Abnorm Child Psychol.

[30] Tully LA, Hunt C (2016). Brief Parenting Interventions for Children at Risk of Externalizing Behavior Problems: A Systematic Review. J Child Fam Stud.

[31] Kaminski J, Valle LA, Filene JH, Boyle CL (2008). A Meta-analytic Review of Components Associated with Parent Training Program Effectiveness. J Abnorm Child Psychol.

[32] Leijten P, Gardner F, Melendez-Torres GJ, van Aar J, Hutchings J, Schulz S, Knerr W, Overbeek G (2019). Meta-Analyses: Key Parenting Program Components for Disruptive Child Behavior. J Am Acad Child Adolesc Psychiatry.

[33] Hornstra R, van der Oord S, Staff AI, Hoekstra PJ, Oosterlaan J, van der Veen-Mulders L, Luman M, van den Hoofdakker BJ (2021). Which Techniques Work in Behavioral Parent Training for Children with ADHD? A Randomized Controlled Microtrial. J Clin Child Adolesc Psychol.

[34] Hornstra R, Groenman AP, van der Oord S, Luman M, Dekkers TJ, van der Veen-Mulders L, Hoekstra PJ, van den Hoofdakker BJ (2022). Review: Which components of behavioral parent and teacher training work for children with ADHD? – a metaregression analysis on child behavioral outcomes.

[35] Nijboer M, van Doornik R, Groenman A, van der Oord S, Hornstra R, van den Hoofdakker B, et al. Brief Behavioural Parent Training for Children with Impairing ADHD Characteristics: a Pilot Study. 2024. Retrieved February 2024 from https://osf.io/preprints/psyarxiv/ahkev. 10.1111/camh.12743 PMC11754698 39644180

[36] Van den Hoofdakker BJ, van der Veen-Mulders L, Sytema S, Emmelkamp PMG, Minderaa RB, Nauta MH (2007). Effectiveness of behavioral parent training for children with ADHD in routine clinical practice: A randomized controlled study. J Am Acad Child Adolesc Psychiatry.

[37] Breen MJ, Altepeter TS (1991). Factor Structures of the Home Situations Questionnaire and the School Situations Questionnaire. J Pediatr Psychol.

[38] Lipszyc J, Schachar R (2010). Inhibitory control and psychopathology: A meta-analysis of studies using the stop signal task. J Int Neuropsychol Soc.

[39] Noordermeer SDS, Luman M, Buitelaar JK, Hartman CA, Hoekstra PJ, Franke B, Faraone SV, Heslenfeld DJ, Oosterlaan J (2020). Neurocognitive Deficits in Attention-Deficit/Hyperactivity Disorder With and Without Comorbid Oppositional Defiant Disorder. J Atten Disord.

[40] Matthys W, Vanderschuren LJMJ, Schutter DJLG, Lochman JE (2012). Impaired Neurocognitive Functions Affect Social Learning Processes in Oppositional Defiant Disorder and Conduct Disorder: Implications for Interventions. Clin Child Fam Psychol Rev.

[41] Johansen EB, Killeen PR, Russell VA, Tripp G, Wickens JR, Tannock R, Williams J, Sagvolden T (2009). Origins of altered reinforcement effects in ADHD. In Behavioral and Brain Functions.

[42] Luman M, Tripp G, Scheres A (2010). Identifying the neurobiology of altered reinforcement sensitivity in ADHD: A review and research agenda. Neurosci Biobehav Rev.

[43] Marsh AA, Blair RJR (2008). Deficits in facial affect recognition among antisocial populations: A meta-analysis. Neurosci Biobehav Rev.

[44] Van der Oord S, Tripp G (2020). How to Improve Behavioral Parent and Teacher Training for Children with ADHD: Integrating Empirical Research on Learning and Motivation into Treatment. Clin Child Fam Psychol Rev.

[45] Abikoff H, Gallagher R, Wells KC, Murray DW, Huang L, Lu F, Petkova E (2013). Remediating organizational functioning in children with ADHD: Immediate and long-term effects from a randomized controlled trial. J Consult Clin Psychol.

[46] Abikoff H, Thompson M, Laver-Bradbury C, Long N, Forehand RL, Miller Brotman L, Klein RG, Reiss P, Huo L, Sonuga-Barke E (2015). Parent training for preschool ADHD: a randomized controlled trial of specialized and generic programs. J Child Psychol Psychiatry.

[47] Sealed Envelope Ltd. Create a blocked randomisation list. 2022. Retrieved September 27, 2022 from https://www.sealedenvelope.com/simple-randomiser/v1/lists.

[48] Eyberg SM, Ross AW (1978). Assessment of child behavior problems: The validation of a new inventory. J Clin Child Psychol.

[49] Abrahamse ME, Junger M, Leijten PHO, Lindeboom R, Boer F, Lindauer RJL (2015). Psychometric Properties of the Dutch Eyberg Child Behavior Inventory (ECBI) in a Community Sample and a Multi-Ethnic Clinical Sample. J Psychopathol and Behav Assess.

[50] Ravens-Sieberer U, Bullinger M (1998). Assessing health-related quality of life in chronically ill children with the German KINDL: first psychometric and content analytical results. Qual Life Res.

[51] Bullinger M, Brütt AL, Erhart M, Ravens-Sieberer U (2008). Psychometric properties of the KINDL-R questionnaire: results of the BELLA study. Eur Child Adolesc Psychiatry.

[52] Shelton KK, Frick PJ, Wootton J (1996). Assessment of parenting practices in families of elementary school-age children. J Clin Child Psychol.

[53] Herbert SD, Harvey EA, Roberts JL, Wichowski K, Lugo-Candelas CI (2013). A Randomized Controlled Trial of a Parent Training and Emotion Socialization Program for Families of Hyperactive Preschool-Aged Children. Behav Ther.

[54] Berry JO, Jones WH (1995). The Parental Stress Scale: Initial Psychometric Evidence. J Soc Pers Relat.

[55] Johnston C, Mash EJ (1989). A Measure of Parenting Satisfaction and Efficacy. J Clin Child Psychol.

[56] Lange A, Evers A, Jansen H, Dolan C (2002). PACHIQ-R: The Parent-Child Interaction Questionnaire-Revised. Fam Process.

[57] EuroQol Research Foundation. EQ-5D-5L UserGuide. 2019. Retrieved December 2023 from https://euroqol.org/publications/user-guides.

[58] Herdman M, Gudex C, Lloyd A, Janssen M, Kind P, Parkin D, Bonsel G, Badia X (2011). Development and preliminary testing of the new five-level version of EQ-5D (EQ-5D-5L). Qual Life Res.

[59] Versteegh MM, Vermeulen KM, Evers SMAA, de Wit GA, Prenger R, Stolk EA (2016). Dutch Tariff for the Five-Level Version of EQ-5D. Value Health..

[60] Bouwmans CAM, Schawo SJ, Jansen DEMC, Vermeulen K, Reijneveld M, Hakkaart-van Roijen L. Handleiding Vragenlijst Intensieve Jeugdzorg: Zorggebruik en productieverlies. Erasmus MC. 2012. Retrieved December 2023 from https://hdl.handle.net/11370/54c0b532-1301-4a38-9094-1936e11343a4.

[61] Zorginstituut Nederland. 2020. Medicijnkosten.nl. 2020. Retrieved December 2023 from https://www.medicijnkosten.nl/.

[62] Drost RMWA, Paulus ATG, Ruwaard D, Evers SMAA (2014). Handleiding intersectorale kosten en baten van (preventieve) interventies. Classificatie, identificatie en kostprijzen.

[63] Kooiman CG, Klaassens ER, van Heloma Lugt JQ, Kamperman AM (2013). Psychometrics and validity of the Dutch Experiences in Close Relationships-Revised (ECR–r) in an outpatient mental health sample. J Pers Assess.

[64] Wei M, Russell DW, Mallinckrodt B, Vogel DL (2007). The Experiences in Close Relationship Scale (ECR)-Short Form: Reliability, Validity, and Factor Structure. J Pers Assess.

[65] Youth in Mind. Self-report SDQ for 17+ years. 2020. Retrieved December 2023 from https://www.sdqinfo.org/py/sdqinfo/b3.py?language=Dutch.

[66] Van den Berg I, Franken IHA, Muris P (2010). A New Scale for Measuring Reward Responsiveness. Front Psychol.

[67] Luman M, van Meel CS, Oosterlaan J, Geurts HM (2012). Reward and Punishment Sensitivity in Children with ADHD: Validating the Sensitivity to Punishment and Sensitivity to Reward Questionnaire for Children (SPSRQ-C). J Abnorm Child Psychol.

[68] Bearss K, Lecavalier L, Minshawi N, Johnson C, Smith T, Handen B, Sukhodolsky DG, Aman MG, Swiezy N, Butter E, Scahill L (2013). Toward an exportable parent training program for disruptive behaviors in autism spectrum disorder. Neuropsychiatry.

[69] Eyberg SM, Johnson SM (1974). Multiple assessment of behavior modification with families: Effects of contingency contracting and order of treated problems. J Consult Clin Psychol.

[70] Breider S, de Bildt A, Nauta MH, Hoekstra PJ, van den Hoofdakker BJ (2019). Self-directed or therapist-led parent training for children with attention deficit hyperactivity disorder? A randomized controlled non-inferiority pilot trial. Internet Interv.

[71] Kostyrka-Allchorne K, Ballard C, Byford S, Daley D, Downs J, French B, et al. Online Parent Training for The Initial Management of ADHD referrals (OPTIMA): the protocol for a randomised controlled trial of a digital parenting intervention implemented to support parents and children on a treatment waitlist. Trials. 2022;23(1):1003. 10.21203/rs.3.rs-2119453/v1. 10.1186/s13063-022-06952-z PMC9744042 36510236

[72] Daley D, van der Oord S, Ferrin M, Danckaerts M, Doepfner M, Cortese S, Sonuga-Barke EJS (2014). Behavioral Interventions in Attention-Deficit/Hyperactivity Disorder: A Meta-Analysis of Randomized Controlled Trials Across Multiple Outcome Domains. J Am Acad Child Adolesc Psychiatry.

[73] Faul F, Erdfelder E, Lang A-G, Buchner A (2007). G*Power 3: A flexible statistical power analysis program for the social, behavioral, and biomedical sciences. Behav Res Methods.

[74] Twisk JWR. Practical guides to biostatistics and epidemiology: Applied multilevel analysis: A practical guide for medical researchers: A practical guide. Cambridge University Press. 2010. 10.1017/cbo9780511610806.

[75] Twisk J, de Boer M, de Vente W, Heymans M (2013). Multiple imputation of missing values was not necessary before performing a longitudinal mixed-model analysis. J Clin Epidemiol.

