# Supplementary material for: The short- and longer-term effects of brief behavioral parent training versus care as usual in children with behavioral difficulties: study protocol for a randomized controlled trial
Source: BMC Psychiatry. 2024 Mar 12;24:203. doi: 10.1186/s12888-024-05649-8 (PMC10936011; doi:10.1186/s12888-024-05649-8)
Supplement: Supplementary file 1 — Supplementary Material 1. [file 12888_2024_5649_MOESM1_ESM.docx]

**Appendix A: Information Letter (in Dutch)**

**
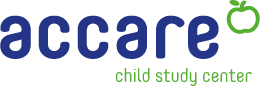

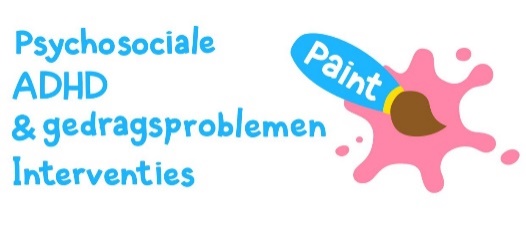
**

**Informatiebrief voor ouder(s)/verzorger(s)**

**PAINT-P: Psychosociale ADHD en Gedragsproblemen Interventies – Oudertraining**

Een onderzoek naar een kortdurende training voor ouders van kinderen met gedragsproblemen.

Beste ouders/verzorgers,

Oudertraining is een goede behandeling voor het verminderen van gedragsproblemen bij kinderen en daarom een belangrijk onderdeel van de zorg die wij bieden. Door onze oudertrainingen goed te onderzoeken, willen we deze steeds beter maken. De meeste oudertrainingen duren vrij lang (gemiddeld 12 sessies). Om ouders en kinderen met gedragsproblemen sneller en beter te kunnen helpen, willen we onderzoeken of oudertraining korter kan. In dit onderzoek bieden we ouders een korte training (“stoomcursus”) waarin ze opvoedingstechnieken leren die ze kunnen gebruiken om het gedrag van hun kind te beïnvloeden.

In deze informatiebrief vindt u informatie over het onderzoek. We willen u vragen de informatie rustig door te lezen. Als u na het lezen nog vragen heeft, kunt u die stellen aan de onderzoeker. U beslist zelf of u wel of niet meedoet aan het onderzoek. Uw keuze heeft geen gevolgen voor het vervolg van de behandeling van uw kind.

Met vriendelijke groeten,

Roos van Doornik, MSc.

Wetenschappelijk onderzoeker

Accare

Prof. dr. Barbara van den Hoofdakker

Wetenschappelijk onderzoeker/klinisch psycholoog

Accare/Rijksuniversiteit Groningen

Dr. Tycho Dekkers

Wetenschappelijk onderzoeker/GZ-psycholoog

Accare

Prof. dr. Saskia van der Oord

Wetenschappelijk onderzoeker/klinisch psycholoog

Katholieke Universiteit Leuven

Dr. Marjolein Luman

Wetenschappelijk onderzoeker/psycholoog

Vrije Universiteit Amsterdam

**Oudertraining bij gedragsproblemen**

Oudertraining is een goede methode om ouders van kinderen met gedragsproblemen te helpen. Met gedragsproblemen bedoelen we bijvoorbeeld druk of impulsief gedrag, driftbuien, niet luisteren of agressief gedrag. Het doel van een oudertraining is om deze problemen te verminderen. In een oudertraining leert u technieken die hiervoor belangrijk zijn. Een behandelaar legt u uit hoe gedragsproblemen kunnen ontstaan en maakt samen met u een plan hoe u met de gedragsproblemen om kunt gaan.

Speciaal voor ons onderzoek hebben we een korte training (stoomcursus) voor ouders ontwikkeld. Deze stoomcursus is veel korter dan de gewone oudertrainingen en bestaat uit drie bijeenkomsten. De bijeenkomsten vinden snel (ongeveer één of twee weken) na elkaar plaats. De eerste twee bijeenkomsten duren ieder twee uur, de derde bijeenkomst duurt één uur. Na afloop van de stoomcursus beoordeelt de behandelaar van uw kind samen met u of verdere hulp nodig is.

Deze verdere hulp kan bestaan uit boostersessies. Boostersessies zijn bijeenkomsten van één uur waarin de behandelaar met u meekijkt hoe u de technieken die u in de stoomcursus hebt geleerd kunt blijven gebruiken. Deze boostersessies kunnen maximaal eens per vier weken worden gegeven tot een jaar na de stoomcursus. De stoomcursus en boostersessies worden gegeven door goed geschoolde behandelaren met veel ervaring met het trainen van ouders van kinderen met gedragsproblemen.

Na de stoomcursus kunnen u en uw kind, eventueel naast de boostersessies, ook gebruik maken van onze reguliere zorg. De reguliere zorg bestaat uit alle zorg die behandelaren ouders en kinderen met gedragsproblemen normaal gesproken aanbieden.

**Voor wie is de stoomcursus bedoeld?**

De stoomcursus is voor ouders van kinderen van 2 tot en met 11 jaar bij wie sprake is van gedragsproblemen in de thuissituatie. We vinden het fijn als beide ouders (als die er zijn) aan de stoomcursus meedoen. Als dit niet lukt, dan kan één ouder meedoen. Uiteraard kunnen ook alleenstaande ouders, pleegouders, adoptieouders en stiefouders aan de stoomcursus meedoen. Als u het afgelopen jaar al een oudertraining hebt gevolgd, kunt u niet meedoen aan het onderzoek. Om mee te kunnen doen aan het onderzoek is het verder van belang dat uw kind geen medicatie voor de gedragsproblemen gebruikt.

**Wat is het doel van het onderzoek?**

Het onderzoek heeft verschillende doelen. Allereerst we willen graag weten of het gedrag van uw kind is veranderd nadat u de stoomcursus en eventueel boostersessies hebt gevolgd. Daarnaast willen we weten wat de stoomcursus met eventuele boostersessies kost en oplevert en dit vergelijken met de reguliere zorg. Ook willen we graag weten hoe verschillende eigenschappen van ouders en kinderen de effecten van de stoomcursus en eventuele boostersessies beïnvloeden en hoe tevreden u als ouder over de stoomcursus bent.

**Hoe onderzoeken we dit?**

In dit onderzoek vergelijken we twee groepen met elkaar. Eén groep ouders volgt de stoomcursus en één groep ouders krijgt de reguliere zorg zoals die wordt geboden op de plek waar uw kind wordt behandeld. Als u deelneemt aan het onderzoek, wordt er geloot in welke groep u meedoet. U kunt dus niet zelf kiezen in welke groep u komt. U heeft evenveel kans om in elk van de twee groepen terecht te komen.

Aan de hand van verschillende vragenlijsten, korte telefoontjes en geluidsopnames zullen we meten hoe het met u en uw kind gaat. Door deze gegevens te vergelijken tussen de groep ouders die de stoomcursus krijgt en de groep ouders die reguliere zorg krijgt, willen we nagaan in hoeverre de nieuwe stoomcursus van meerwaarde is. Omdat we het belangrijk vinden wat ouders van de stoomcursus vinden, zullen enkele ouders na afloop ook uitgenodigd worden deel te nemen aan een klankbordgroep (één bijeenkomst).

**Wat verwachten we van u?**

***Vragenlijsten***

Als u meedoet aan het onderzoek vragen we u een paar vragenlijsten thuis op de computer in te vullen. Als u deze vragenlijsten liever op papier of telefonisch invult, kan dit ook. Dit zijn vragenlijsten die op sommige plekken ook in de reguliere zorg (dus buiten dit onderzoek) gebruikt worden. U wordt gevraagd deze vragenlijsten voor de training, direct na de training, een halfjaar na de training en een jaar na de training in te vullen. Het invullen van de vragenlijsten duurt ongeveer 30 tot 45 minuten per keer.

***Korte telefoontjes***

We willen het gedrag van uw kind graag goed volgen. Daarom zullen we vaak telefonisch contact opnemen om te horen hoe het gaat. Vijf aaneensluitende dagen voordat de training begint, vijf aaneensluitende dagen direct na de training, vijf aaneensluitende dagen een halfjaar na de training en vijf aaneensluitende dagen een jaar na de training krijgt u elke dag een kort telefoontje (ongeveer twee minuten) op een vooraf afgesproken tijdstip. Iemand van het onderzoeksteam zal u dan bellen om te horen hoe het gedrag van uw kind die dag verlopen is. Zo kunnen we goed nagaan of de stoomcursus helpt om het gedrag van uw kind te veranderen.

***Geluidsopnames***

Om het gedrag van uw kind nog beter te volgen, vragen we u ook om voor en na de training geluidsopnames te maken tijdens de maaltijd. Maaltijden zijn vaak momenten waarop kinderen verschillende gedragsproblemen laten zien. Deze opnames kunnen ons daarom goed helpen om na te gaan of de stoomcursus helpt. Bij deze opnames wordt alleen het geluid opgenomen en dus **geen** beeld. U wordt gevraagd deze geluidsopnames voor de training, direct na de training, en een jaar na de training te maken. Deze geluidsopnames worden op een beveiligde server opgeslagen. Alleen de onderzoekers zelf kunnen in deze server komen. Uw behandelaar zal deze geluidsopnames dus **niet** te horen krijgen. En u kunt de onderzoekers altijd vragen of u de geluidsopnames mag naluisteren of, als dit nodig is, om de opnames te verwijderen.

Daarnaast zal de behandelaar geluidsopnames van de stoomcursus maken. Deze zijn uitsluitend bedoeld om precies te kunnen controleren wat de behandelaar met u besproken heeft. We zullen hiervan dus geen informatie gebruiken over uzelf of uw kind.

***Dossier***

Als u meedoet aan het onderzoek, willen de onderzoekers het dossier van uw kind bekijken om diagnostische gegevens in te zien en na te gaan wanneer welke zorg is aangeboden. Zo kunnen we de kosten en opbrengsten van verschillende vormen van zorg goed met elkaar vergelijken. De gegevens die de onderzoekers inzien, worden alleen voor het onderzoek gebruikt en **niet** met anderen gedeeld.

**Voor- en nadelen van deelname aan dit onderzoek**

- U krijgt, als u wordt ingeloot voor de korte oudertraining, een stoomcursus waarin u verschillende technieken leert om het probleemgedrag van uw kind te verminderen. De training kost u (veel) minder tijd dan een gewone oudertraining.
- De stoomcursus wordt gegeven door goed geschoolde behandelaren met veel ervaring met (ouders van) kinderen met gedragsproblemen.
- Als u niet wordt ingeloot voor de korte oudertraining, krijgt u de reguliere zorg die behandelaren ouders en kinderen met gedragsproblemen normaal gesproken aanbieden.
- U helpt andere ouders en kinderen met gedragsproblemen. Door het onderzoek leren we namelijk hoe we oudertrainingen kunnen verbeteren. Dit kan andere ouders en kinderen in de toekomst helpen.
- U vult vragenlijsten in en maakt geluidsopnames. Dit kost u iets meer tijd dan wanneer u niet aan het onderzoek meedoet.
- Tijdens de looptijd van het onderzoek wordt u in vier periodes gedurende vijf dagen elke dag op een afgesproken tijdstip kort gebeld. Door middel van deze dagelijkse telefoontjes wordt nauwlettend in kaart gebracht hoe het met uw kind gaat. De telefoontjes duren ongeveer twee minuten per keer.
- Tijdens de stoomcursus kunnen u en uw kind in principe geen gebruik maken van andere, reguliere, zorg voor de gedragsproblemen, tenzij dit noodzakelijk is voor de gezondheid van u of uw kind. Direct na de stoomcursus (na ongeveer vier weken) kunt u gebruik maken van de boostersessies en de reguliere zorg.

**Wat gebeurt er als u niet mee wilt doen?**

U beslist zelf of u meedoet aan het onderzoek. Als u niet meedoet, heeft dit geen gevolgen voor de verdere behandeling van uw kind. U krijgt dan de gebruikelijke zorg aangeboden, uw behandelaar kan u uitleggen wat dit inhoudt. Aan de stoomcursus kunt u alleen deelnemen als u meedoet aan het onderzoek. U hoeft geen reden op te geven als u niet aan het onderzoek wilt meedoen. Ook als u wel meedoet, kunt u op elk moment stoppen zonder daarvoor een reden te geven.

**Wat gebeurt er als het onderzoek is afgelopen?**

Uw deelname aan het onderzoek stopt als alle metingen voorbij zijn of als u zelf kiest om eerder te stoppen. Na de stoomcursus bekijkt u samen met de behandelaar of het gedrag van uw kind is verbeterd en of verdere behandeling of ondersteuning nog nodig is. Het hele onderzoek is afgelopen als alle deelnemers klaar zijn. Als u wilt, kunt u na afloop van het onderzoek informatie krijgen over de belangrijkste uitkomsten van het onderzoek. Deze informatie kan worden gegeven wanneer alle deelnemers klaar zijn en alle gegevens zijn verwerkt.

**Wat kost het om mee te doen aan het onderzoek?**

Meedoen aan dit onderzoek kost u niets.

**Wat gebeurt er met de gegevens van u en uw kind?**

Om uw privacy te beschermen, blijven de gegevens die we over u en uw kind verzamelen geheim. De gegevens bewaren we onder een code en niet onder de naam van u of uw kind. Als we bekijken wat de resultaten van het onderzoek zijn, gebeurt dat altijd met deze codes. De onderzoekers die betrokken zijn bij het onderzoek, kunnen als enige nagaan welke code bij welke persoon hoort. Alle persoonsgegevens en gecodeerde onderzoeksgegevens worden op een beveiligde server opgeslagen. De sleutel tussen persoonsgegevens en gecodeerde onderzoeksgegevens wordt in een apart beveiligd bestand opgeslagen. De onderzoekers zijn wettelijk verplicht om uw gegevens minimaal 15 jaar te bewaren.

Het is eventueel mogelijk dat de onderzoekers u na dit onderzoek willen benaderen voor vervolgonderzoek, of dat zij de gegevens die voor dit onderzoek zijn verzameld willen gebruiken voor vervolgonderzoek. Door het toestemmingsformulier te ondertekenen, geeft u toestemming voor deze mogelijkheid. Uw toestemming draagt bij aan het uitvoeren van wetenschappelijk onderzoek, maar is uiteraard geheel vrijwillig.

**Hoe kunt u meedoen?**

Als u (misschien) wilt meedoen aan het onderzoek, kunt u dat met uw behandelaar bespreken. U geeft dan bij uw behandelaar aan dat de onderzoekers u mogen bellen. De onderzoekers krijgen vervolgens via de behandelaar uw naam en contactgegevens. De onderzoekers zullen u dan zo snel mogelijk bellen om verdere uitleg te geven over het onderzoek en uw eventuele vragen te beantwoorden.

Om definitief mee te kunnen doen aan het onderzoek is het nodig dat de alle ouders met ouderlijk gezag/wettelijk vertegenwoordigers van het kind een toestemmingsformulier ondertekenen. U heeft 14 dagen de tijd om te bedenken of u mee wilt doen aan het onderzoek. Wilt u er langer over nadenken, dan kunt u dit aangeven. Ook bij vragen, twijfels of opmerkingen kunt u contact opnemen met de onderzoekers.

**Contact**

Wilt u direct contact? Dan kunt u bellen of mailen met:

Roos van Doornik, MSc.

Wetenschappelijk onderzoeker (promovenda)

Accare Child Study Center

Tel: 06-59820639

Mail: r.van.doornik@accare.nl

Postadres: Accare Child Study Center

T.a.v. R. van Doornik

Postbus 660

9700 AR Groningen
