# Supplementary material for: The short- and longer-term effects of brief behavioral parent training versus care as usual in children with behavioral difficulties: study protocol for a randomized controlled trial
Source: BMC Psychiatry. 2024 Mar 12;24:203. doi: 10.1186/s12888-024-05649-8 (PMC10936011; doi:10.1186/s12888-024-05649-8)
Supplement: Supplementary file 2 — Supplementary Material 2. [file 12888_2024_5649_MOESM2_ESM.docx]

**Appendix B: Informed Consent Form (in Dutch)**


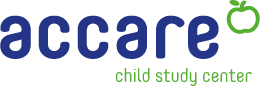

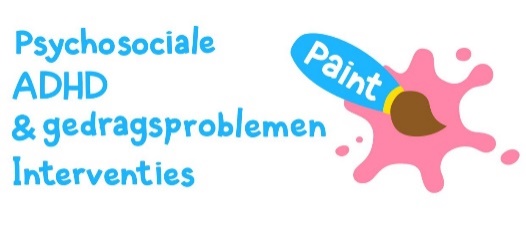


**Toestemmingsverklaring voor ouder(s)/verzorger(s)**

Verantwoordelijke onderzoekers Naam: Prof. dr. Barbara van den Hoofdakker

Email: [b.van.den.hoofdakker@accare.nl](mailto:b.van.den.hoofdakker@accare.nl)

Naam: Dr. Marjolein Luman

Email: [m.luman@vu.nl](mailto:m.luman@vu.nl)

Uitvoerende onderzoeker Naam: Roos van Doornik, Msc.

Email: [r.van.doornik@accare.nl](mailto:r.van.doornik@accare.nl)

Telnr: 06-59820639

Geachte ouder/verzorger,

Wilt u bijgaand formulier goed doorlezen en ondertekenen als u akkoord bent met alle genoemde punten?

Ik ben gevraagd om toestemming te geven voor deelname aan het wetenschappelijk onderzoek:

“PAINT-P: Psychosociale ADHD en Gedragsproblemen Interventies – Oudertraining”.

**Ik verklaar dat**

- ik de informatiebrief heb gelezen;
- ik aanvullende vragen kon stellen en deze vragen naar tevredenheid zijn beantwoord;
- ik goed heb begrepen wat deelname aan het onderzoek inhoudt;
- ik voldoende tijd heb gehad om te beslissen om mee te doen;
- ik weet dat meedoen aan het onderzoek helemaal vrijwillig is;
- ik weet dat ik op ieder moment kan beslissen om toch niet mee te doen aan het onderzoek en dat ik daarvoor dan geen reden op hoef te geven;
- ik weet dat sommige bij het onderzoek betrokken mensen de gegevens van mij en mijn kind kunnen zien, zoals in de informatiebrief staat;
- Ik weet dat alle gegevens vertrouwelijk zullen worden behandeld en dat resultaten van het onderzoek alleen anoniem aan derden bekend gemaakt zullen worden, zoals in de informatiebrief staat.

**Ik geef toestemming**

- voor deelname aan dit onderzoek;
- om de gegevens over mij en mijn kind te verzamelen en gebruiken voor het onderzoek op de wijze waarop dat in de informatiebrief staat beschreven, de onderzoekers doen dit alleen om de onderzoeksvragen van dit onderzoek te beantwoorden;
- om de gecodeerde onderzoeksgegevens van mij en mijn kind tot minimaal 15 jaar na afloop van het onderzoek te bewaren, zoals in de informatiebrief staat beschreven;
- om tijdens het onderzoek gebruik te maken van het medisch dossier van mijn kind om gegevens over de diagnostiek en behandeling te verzamelen;
- om de gecodeerde onderzoeksgegevens in de toekomst te gebruiken voor vervolgonderzoek.

Ja / Nee* **doorhalen wat niet van toepassing is*

Naam kind: ____________________________________

Geboortedatum: _______ / _______ / _______

Naam en handtekening van **alle** ouders met ouderlijk gezag / wettelijk vertegenwoordigers van het kind:

1. Naam: ____________________________________

Handtekening: ____________________________________

Datum: _______ / _______ / _______

1. Naam: ____________________________________

Handtekening: ____________________________________

Datum: _______ / _______ / _______

***Onderstaand gedeelte hoeft u niet in te vullen. Dat is bestemd voor de onderzoeker.***

Ik verklaar hierbij dat ik bovengenoemde persoon/personen heb geïnformeerd over het genoemde onderzoek. Als er tijdens het onderzoek informatie bekend wordt die de toestemming van de ouder(s) of verzorgers kunnen beïnvloeden, dan breng ik hem/haar daarvan tijdig op de hoogte.

Naam onderzoeker: ____________________________________

Handtekening: ____________________________________

Datum: _______ / _______ / _______
