# Supplementary figures and images for: The short- and longer-term effects of brief behavioral parent training versus care as usual in children with behavioral difficulties: study protocol for a randomized controlled trial
Source: BMC Psychiatry. 2024 Mar 12;24:203. doi: 10.1186/s12888-024-05649-8 (PMC10936011; doi:10.1186/s12888-024-05649-8)

**Appendix C: Schedule of Enrolment, Interventions and Assessments**


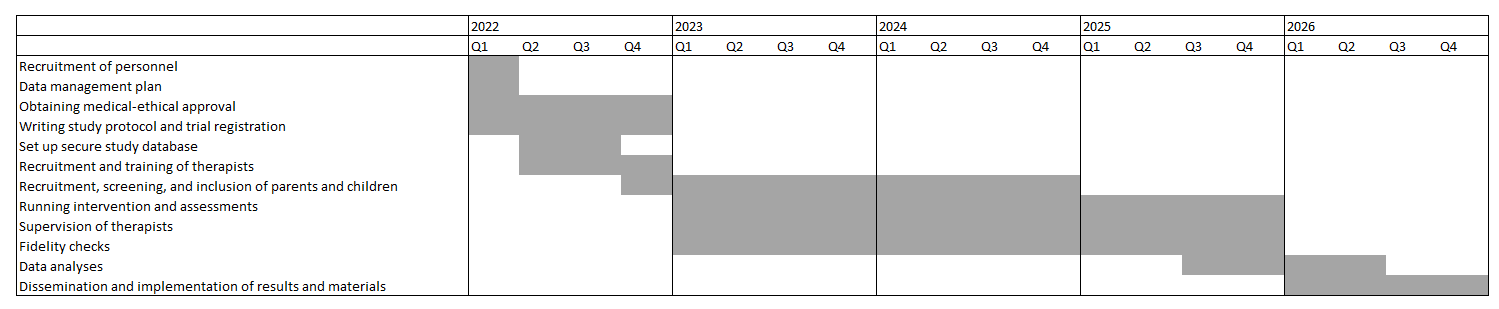

Supplement: Supplementary file 3 — Supplementary Material 3. [file 12888_2024_5649_MOESM3_ESM.docx]
